# Effects of dietary energy levels on rumen fermentation, microbiota, and gastrointestinal morphology in growing ewes

**DOI:** 10.1002/fsn3.1955

**Published:** 2020-11-10

**Authors:** Qiye Wang, Yancan Wang, Xin Wang, Chunpeng Dai, Wensheng Tang, Jianzhong Li, Pengfei Huang, Yali Li, Xueqin Ding, Jing Huang, Tarique Hussain, Huansheng Yang, Mingzhi Zhu

**Affiliations:** ^1^ Hunan Provincial Key Laboratory of Animal Intestinal Function and Regulation Hunan International Joint Laboratory of Animal Intestinal Ecology and Health Laboratory of Animal Nutrition and Human Health College of Life Sciences Hunan Normal University Changsha Hunan China; ^2^ Hubei Zhiqinghe Agriculture and Animal Husbandry Co., Ltd Yichang Hubei China; ^3^ Animal Sciences Division Nuclear Institute for Agriculture and Biology (NIAB) Faisalabad Pakistan; ^4^ Key Laboratory of Agro‐ecological Processes in Subtropical Region Hunan Provincial Engineering Research Center of Healthy Livestock Scientific Observing and Experimental Station of Animal Nutrition and Feed Science in South‐Central Ministry of Agriculture Institute of Subtropical Agriculture Chinese Academy of Sciences Changsha Hunan China; ^5^ National Research Center of Engineering Technology for Utilization of Functional Ingredients from Botanicals Co‐Innovation Center of Education Ministry for Utilization of Botanical Functional Ingredients College of Horticulture Hunan Agricultural University Changsha China

**Keywords:** Gastrointestinal tract, High‐throughput sequencing, Rumen microbiota, Volatile fatty acid

## Abstract

This study investigated whether dietary metabolizable energy (ME) could generate dynamical effects on rumen fermentation, gastrointestinal tract (GIT) morphology, and microbial composition of growing ewes. A total of twenty‐eight female Hu lambs were randomly allotted to two treatments with different dietary ME levels: 9.17 (FEA) and 10.41 MJ/kg (FEB). These lambs were further made ready for a 67‐day feeding trial. Results showed that the molar proportions of butyrate (*p* = .020), iso‐valerate (*p* = .028), and valerate (*p* = .005) were significantly higher in the FEB group than those in the FEA group. The results of the GIT morphologic properties showed that the villus height (VH) (*p* = .005) was significantly higher and crypt depth was significantly deeper (CD) (*p* = .005) in the duodenum and that the rumen papillary height (PH) was significantly higher (*p* = .020) in FEB group compared with the FEA group. High‐throughput sequencing results showed that 1826 operational taxonomic units (OTUs) were obtained and that the OTU number (*p* = .039), the ACE (*p* = .035), and Chao1 indices (*p* = .005) were lower in the FEB group. Moreover, 76 genera belonging to 21 phyla were detected in all samples; the relative abundance of *Papillibacter* (*p* = .036) and *Flexilinea* (*p* = .046) was significantly lower in the high energy group, whereas the relative abundance of *unidentified Lachnospiraceae* (*p* = .019), *Acetitomaculum* (*p* = .029), *unidentified* V*eillonellaceae* (*p* = .017), *Anaerovibrio* (*p* = .005), and *Succinivibrio* (*p* = .035) was significantly higher in the FEB group at the genus level. Furthermore, the relative abundance of genes and metabolic pathways were predicted by PICRUSt. The relative abundance of gene families related to carbohydrate metabolism was particularly higher (*p* = .027) in the FEB group. In summary, these results reveal that the dietary energy levels altered the composition and function of rumen microbiota and GIT morphology in growing female *Hu* sheep and provide a reference for optimizing diet formula and 10.41MJ/kg of ME level has been recommended in the growing period.

## Graphical abstract

Dietary energy levels can affect the composition and function of rumen microflora and gastrointestinal tract morphology in Hu sheep. Appropriate dietary energy levels are beneficial to the gastrointestinal health of growing ewes in production.

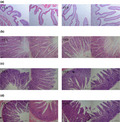

## 1 INTRODUCTION

The *Hu* sheep are known for their high adaptability and reproductive ability in China. Prolific *Hu* sheep are considered a very good maternal resource in the current intensive and factory farming (Lv et al., 2020; Wang, et al., 2020). Diet is a critical determinant factor that influences the composition and function of the rumen microbiome, molar concentration, and proportion of volatile fatty acids (VFAs) and gastrointestinal morphology in ruminants (Carberry et al., 2012; Lee et al., 2012; Wang et al., 2012; da Silva et al., 2020). With the transformation of ruminant farming patterns, the diets are witnessing a predictable change. The rumen micro‐ecosystem and gastrointestinal development have altered accordingly, which ultimately lead to performance traits such as growth and fertility change (Morandfehr et al., 2007; Wang, et al., 2017b; Wang, et al., 2020). Bacteria is the dominant microorganism group in the rumen micro‐ecosystem. Some of which are attached to feed particles can transform the plant ingredients into animal products (Han et al., 2015; Knoell et al., 2016; Pitta et al., 2016). Microbial activity is mostly limited by dietary nutrition, and energy and protein, particularly, are the two determinants (Clark, 1975; Clark & Davis, 1980). For instance, a high energy diet can stimulate the synthesis of microbial proteins by providing sufficient available energy for microbial growth (Bach et al., 2005; Owens et al., 2016). The rumen is the main site for carbohydrate digestion, in which feed ingredients are fermented by rumen microbes into VFAs and absorbed by the GIT (Cunha et al., 2011; McGovern et al., 2018).

Applying high energy diets to improve ruminant performance has been a popular strategy in intensive production, for example, by increasing the proportion of concentrate and using high grain (corn) diets. Fernando et al. (2010) find that feeding a high grain diet causes accelerated accumulation of the VFA and alters the composition and function of the rumen bacterial community. Further studies prove that long‐term feeding of a high grain diet alters the ruminal fermentation and the rumen and ruminal epithelium‐associated microbiome and cause rumen damage and metabolic disorders in cows, goats, and sheep (Hua et al., 2017; Saleem et al., 2012). Impaired rumen and intestinal epithelium can definitely affect the digestion and absorption of VFAs for the GIT. Therefore, keeping rumen fermentation normal and stable, the bacterial community and gastrointestinal morphology are crucial to guarantee the ruminant health and production capacity. Thus, we hypothesize that sheep growth rate and reproductivities may mostly depend on adequate nutrition, the appropriate dietary energy level may directly determine the production level of *Hu* sheep. However, the relationship between rumen microbial community, GIT development, and feed efficiency remains to be elucidated further in *Hu* sheep; Whether dietary energy levels have a dynamic effect on rumen fermentation, gastrointestinal morphology, and rumen bacteria in prebreeding ewes needs further investigation. The aim of this study was to systematically explore the effects of different dietary energy levels on the structural properties of the GIT and the rumen microbial diversity and predicted function in candidate ewes and recommend an appropriate dietary energy level in growing ewes.

## 2 MATERIALS AND METHODS

### 2.1 Ethical statements

The experimental procedures of this study were approved by the Animal Care Committee of Hunan Normal University with reference from the Administration of Affairs Concerning Experimental Animals.

### 2.2 Animals, treatments, and sampling

Twenty‐eight four‐month‐old female Hu lambs with the average initial body weight (IBW) of 18.43 ± 0.34 kg were chosen and randomly allotted to two dietary treatments: 9.17 MJ/kg (FEA) and 10.41MJ/kg (FEB) of ME. Experimental diets met the nutritional requirements (NY/T 816–2004 and NRC, 2007) for prebreeding ewes; The feed composition and nutritional ingredients are shown in Table 1. Feeding and management were implemented after referring to Wang, et al. (2020). Each group was fed in an individual pen with free feeding and automatic water supply. All experimental lambs were fed twice a day at 7:00 and 16:00 hr. The preliminary feeding period was 7 days, and the fixed trial period lasted 60 days. Five lambs with body weight closest to the group's average body weight were selected for euthanasia after 12 hr of fasting from the two treatments according to veterinary police rules at the end of the experiment. The rumen was separated from each sheep, and approximately 100 ml of rumen content was collected and transferred into two 50 ml sterile plastic tubes and then immediately stored at −80°C. A 1 × 1 cm section of the rumen wall was cut, approximately 2 cm long intestinal tissues (duodenum, jejunum, and ileum) from the middle sections were isolated and flushed with ice‐cold phosphate‐buffered saline, and then all the samples were immediately fixed in 4% neutral formalin for morphometric analysis (Yin et al., 2020).

**Table 1:** Diet ingredients and nutrition levels

Item	Dietary treatment	
	FEA (9.17 MJ/kg of ME)	FEB (10.41 MJ/kg of ME)
Ingredient, %		
Corn silage	40	17
Peanut seedling	30	30
Corn	5.44	30.30
Wheat bran	6.96	6.69
Soybean meal	14.60	13.01
Premix^a^	3	3
Total	100	100
Nutrient levels^b^		
Dry matter, g/kg	884	894
Crude fat, g/kg	20	22
Neutral‐detergent fiber, g/kg	453	373
Acid‐detergent fiber, g/kg	332	254
Crude ash, g/kg	67	57
Acid insoluble ash, g/kg	14	10
Crude protein, g/kg	132	129
Non‐fiber carbohydrate^c^, g/kg	314	409
ME, MJ/kg	9.17	10.41

^a^ Premix provides the following per kg: vitamin A 120KIU; vitamin D_3_ 60KIU; vitamin E 200mg; Cu 0.15g; Fe 1g; Zn 1g; Mn 0.5g; I 15mg; Se 5mg; Co 2.5mg; Ca 20g; NaCl 100‐250g; P 10g.

^b^ Except for ME was the predicted value, the rest were measured value.

^c^ Non‐fiber carbohydrate = 1,000 − Neutral‐detergent fiber − Crude protein − Crude fat – Ash.

### 2.3 Rumen fermentation parameters

The VFA concentration was determined by gas chromatography (Agilent 7890A, NYSE: A, Palo Alto, America) according to the method of Wang, et al. (2017). The processing of rumen fluid has been described in Supplementary Material S1 (Section S1.1). Processed samples were automatically injected into an Agilent DB‐FFAP gas‐phase capillary column (30 m × 0.25 mm × 0.25 µm). The injector temperature was set at 250°C and the detector temperature at 280°C. The split ratio of all samples was set at 50:1. The column temperature was heated by programing from 60°C to 220°C with a rate of 20°C/min and followed by holding for a 5 min.

### 2.4 Rumen and intestinal morphology

The rumen and intestinal morphology were analyzed by hematoxylin–eosin (HE) staining and optical microscopy. Paraffin sections of tissues were prepared by referring to the method described by Deng et al. (2020) and Wang et al. (2020a, 2020b). Formalin‐fixed rumen, duodenum, jejunum, and ileum samples were dehydrated and embedded in paraffin, and then, cross‐sections of 5‐μm thickness were cut and stained with HE. The morphological structure of villus height (VH), villus width (VW), crypt depth (CD), and papillary height (PH) was acquired by a microscope using an image processing and analysis system (Version 1, Leica Imaging Systems Ltd., Cambridge, UK). At least ten well‐oriented intact villi and their corresponding crypts were blindly measured by the Image‐Pro Plus 6.0 software in each rumen and intestinal section of each female *Hu* lamb, the VH to CD ratio (VH/CD) was also calculated.

### 2.5 DNA extraction and amplification of 16S rRNA genes

The total microbial genomic DNA was extracted using the CTAB/SDS method. V4 regions of 16S rRNA genes were amplified with forward primer V515F (5′‐ GTGYCAGCMGCCGCGGTAA‐3′) and reverse primer V806R (5′‐GGACTACHVGGGTWTCTAAT‐3′) (Sáenz et al., 2019). The PCR reactions were performed in 30µl systems. The PCR amplified procedure is provided in Supplementary Material S1. The Ion Plus Fragment Library Kit 48 rxns (Thermo Scientific) was used to construct the sequencing libraries, and the Ion S5^TM^ XL platform was used to sequence the library and generate 400‐bp/600‐bp single‐end reads.

### 2.6 Sequencing and bioinformatics analysis

The raw reads were cleaned by the Cutadapt quality control process (Martin, 2011), using the UCHIME algorithm (Edgar et al., 2011) to detect and remove the chimera sequences and finally obtain the clean reads. Sequence analysis was performed by the UPARSE software (UPARSE v7.0.1001) (Edgar, 2013) to cluster the operational taxonomic units (OTUs) with ≥ 97% similarity. Silva Database (Quast et al., 2013) was used to annotate taxonomic information and normalize the abundant OTUs. The alpha diversity and beta diversity were analyzed subsequently by QIIME (Version1.7.0) and displayed by the R Software (Version 2.15.3). Phylogenetic Investigation of Communities by Reconstruction of Unobserved States (PICRUSt) was utilized to predict the metabolic function of the microflora. The raw sequencing data of this study were submitted to the Sequence Read Archive (SRA) with the accession number SRR11996724‐ SRR11996733.

### 2.7 Statistical analysis

The experimental data were analyzed using the one‐way ANOVA in SPSS 18.0 software packages (SPSS, Chicago, IL, USA). The final results were presented with meaning values. Differences were considered to be tendency at 0.05 < *p* < .1 and statistically significant at *p* ≤ .05.

## 3 RESULTS

### 3.1 Rumen fermentation parameters

The results of rumen fermentation parameters are shown in Table 2. The molar proportion of butyrate (*p* = .020), iso‐valerate (*p* = .028), and valerate (*p* = .005) was significantly higher in FEB group than that in FEA group.

### 3.2 Rumen and intestinal morphology

**FIGURE 1:**
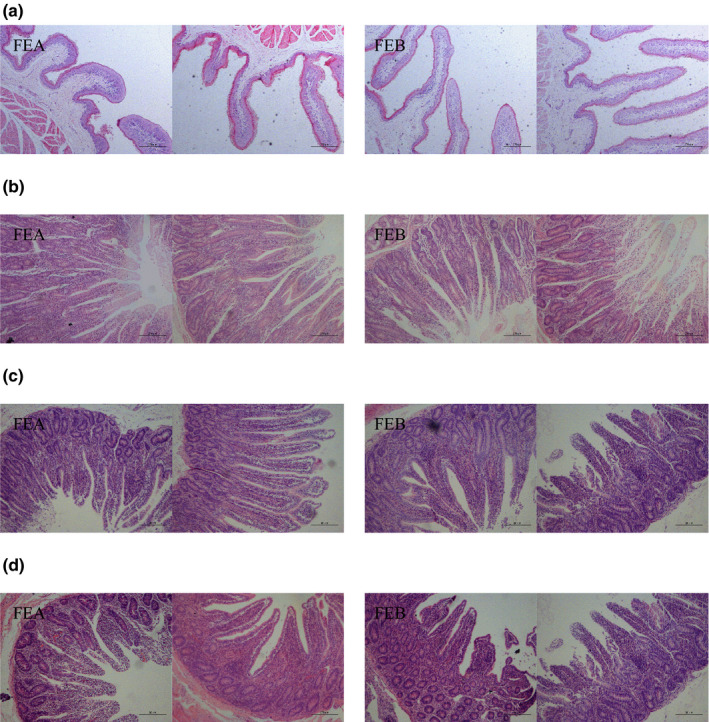
The rumen and small intestinal morphology structure of female lambs with different dietary ME levels (FEA,9.17 MJ/kg of ME and FEB,10.41 MJ/kg of ME). (a) Papillary height of rumen. Scale bar = 500μm. (b) The morphology structure of duodenum. Scale bar = 200μm. (c) The morphology structure of jejunum. Scale bar = 20μm. (d) The morphology structure of ileum. Scale bar = 20μm

The rumen and intestinal morphologic properties are shown in Table 3 and Figure 1. Compared with the FEA group, the VH (*p* = .005) and CD (*p* = .005) were significantly higher in the duodenum, and the VW and VH/CD had not significantly difference in the FEB group. In the jejunum, the VH (*p* = .099) and VH/CD (*p* = .077) tended to decrease, whereas the CD and VW had no significant difference in the FEB group. In the ileum, there were no significant differences in VH, CD, VW, and VH/CD. The rumen PH was significantly higher in the FEB group than that in the FEA group (*p* = .020).

### 3.3 Sequences across different diets

Sequencing analysis showed that a total of 1826 OTUs were obtained at 97% identity. Among them, 1731 (94.80%) OTUs were annotated at the phylum level and 419 (22.95%) OTUs at the genus level (Table S1). Compared with the FEA group, the OTU number (*p* = .039), the richness estimators of ACE (*p* = .035), and Chao1 indices (*p* = .005) were significantly lower in the FEB group. Goods‐coverage indices were 99%, indicating that the sequencing depth could accurately reflect the microbial community for all samples (Table 4).

### 3.4 Composition of the rumen bacterial community

**FIGURE 2:**
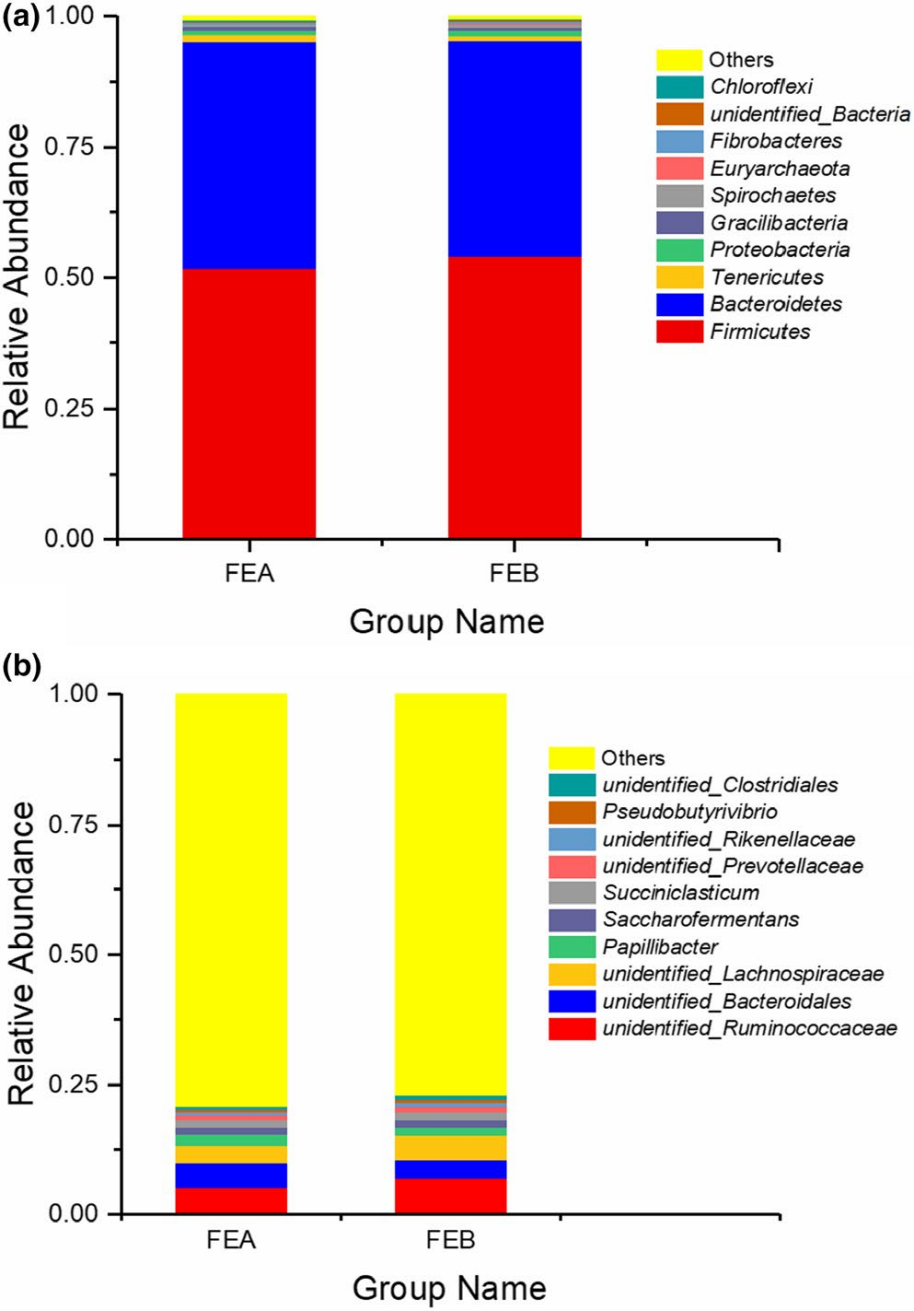
Effects of different dietary energy levels on the composition of the rumen bacterial community. (a) Rumen bacterial communities at the phylum level. (b) The relative abundances of the most dominant genera. A color‐coded bar plot showing the distributions of the bacterial phyla among groups with different dietary energy levels (FEA,9.17 MJ/kg of ME and FEB,10.41 MJ/kg of ME)

A total of 21 phyla were identified by taxonomic analysis, and there were other unclassified bacteria. The five phyla that were most abundant were *Firmicutes*, *Bacteroidetes*, *Tenericutes*, *Proteobacteria,* and *Gracilibacteria* (Figure 2a and Table S2). In the FEA group, the dominant phyla were *Firmicutes* (abundance of 51.71%), *Bacteroidetes* (43.50%), *Tenericutes* (1.28%), *Proteobacteria* (0.88%), and *Gracilibacteria* (0.61%). In the FEB group, *Firmicutes*, *Bacteroidetes*, *Proteobacteria*, *Tenericutes,* and *Spirochetes* were the most abundant phyla, representing 54.13%, 41.14%, 1.10%, 1.00%, and 0.61% of the total reads, respectively. Notably, the relative abundance read of *Firmicutes* and *Bacteroidetes* was the richest in the two tested groups, accounting for more than 95% of the total abundance. Additionally, the relative abundance of *Chloroflexi* (*p* = .041) was considerably lower in the FEB group than that in the FEA group at the phyla level (Table S2).

A total of 76 bacterial genera were detected at the genus level. The ten most abundant genera, which might relate to the most important bacteria affecting the rumen ecosystem, are elucidated in Table S3 and Figure 2b for all the samples. Between the two groups, the ten dominant genera were *unidentified Ruminococcaceae*, *unidentified Bacteroidales*, *unidentified Lachnospiraceae*, *Papillibacter*, *Saccharofermentans*, *Succiniclasticum*, *unidentified Prevotellaceae*, *unidentified Rikenellaceae*, *Pseudobutyrivibrio*, and *unidentified Clostridiales*. Among these genera, *unidentified Ruminococcaceae* belongs to *Firmicutes* in the phylum, *unidentified Bacteroidales* to *Bacteroidetes*, and *unidentified Lachnospiraceae* to *Firmicutes*.

**FIGURE 3:**
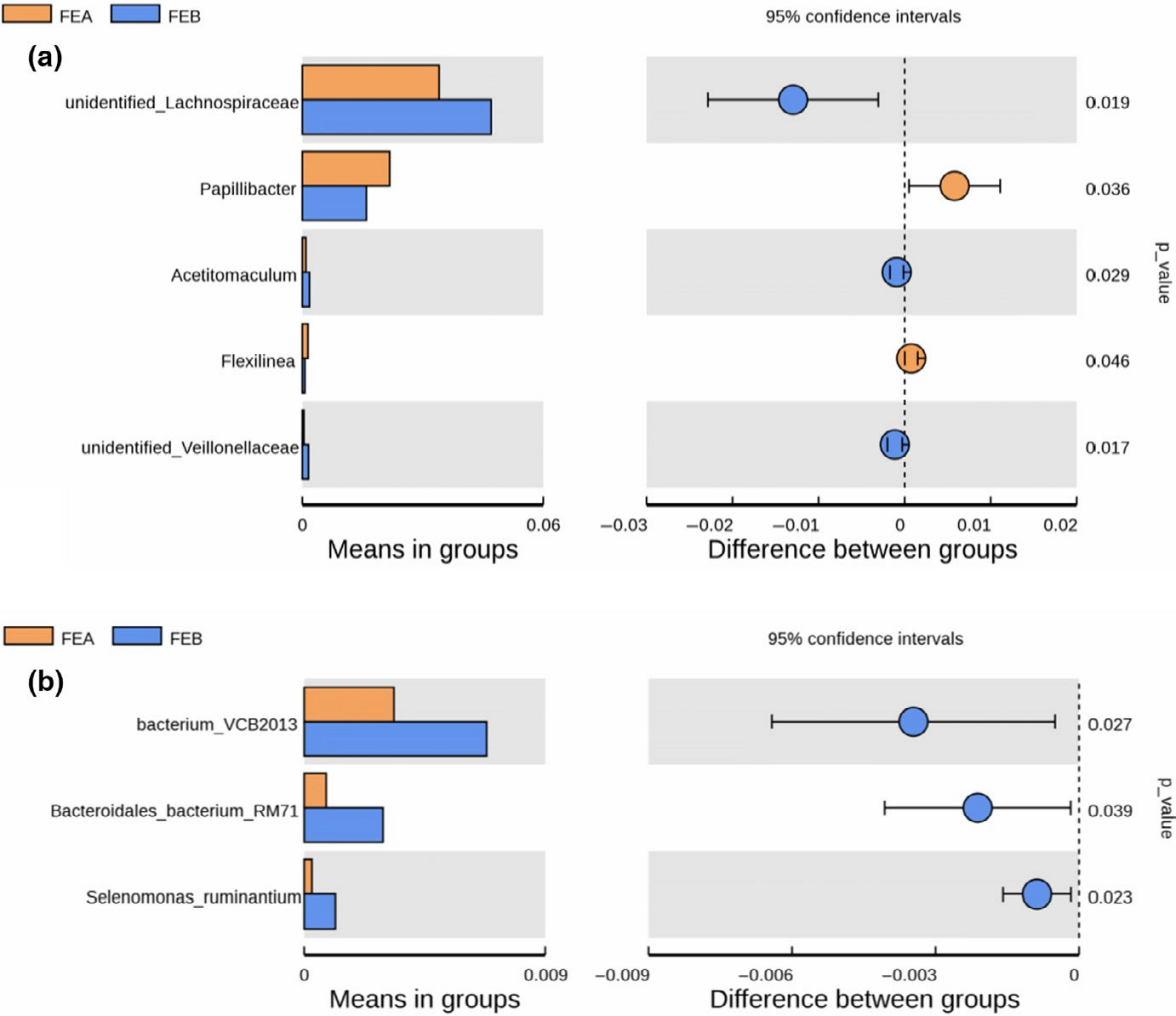
Abundance maps of bacteria with significant differences in (a) genus and (b) species of female Hu lambs fed the low energy diet (FEA,9.17 MJ/kg of ME) and high energy diet (FEB,10.41 MJ/kg of ME)

The relative abundance of *Papillibacter* (*p* = .036) and *Flexilinea* (*p* = .046) in the low energy group (FEA) was significantly higher than that in high energy group (FEB), while the relative abundance of *unidentified Lachnospiraceae* (*p* = .019), *Acetitomaculum* (*p* = .029), *unidentified Veillonellaceae* (*p* = .017), *Anaerovibrio* (*p* = .005), and *Succinivibrio* (*p* = .035) in the low energy group was significantly lower than that in the high energy group at the genus level (Figure 3a and Table S2). Additionally, the relative abundance of *bacterium_VCB2013* (*p* = .027), *Bacteroidales*_*bacterium*_*RM71* (*p* = .039), and *Selenomonas ruminantium* (*p* = .023) in the high energy group was significantly higher than that in the low energy group at the species level (Figure 3b).

### 3.5 Clustering dissimilarities of rumen microbes

**FIGURE 4:**
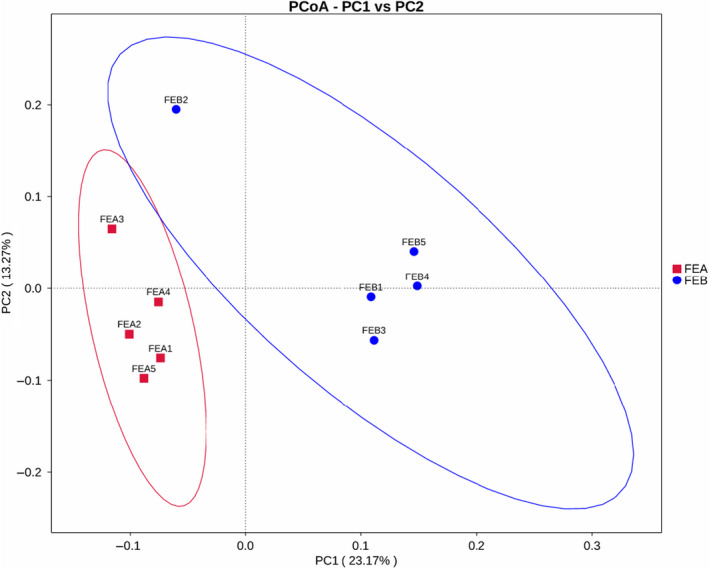
PCoA of rumen microbial community of different dietary energy levels based on unweighted UniFrac distances. Individual samples from FEA1 to FEA5 in group FEA (red,9.17 MJ/kg of ME), FEB1 to FEB5 in group FEB (blue,10.41 MJ/kg of ME). PC = percent variation explained by the axis

The results of principal coordinate analysis (PCoA) showed that the ruminal bacterial communities accounted for 23.17% of the total variations and were obviously distinguished from groups FEA and FEB by PC1, and the bacterial communities between groups FEA and FEB were distinguished and represented 13.27% of the total variation by PC2 (Figure 4). The results of the nonmetric multidimensional scaling (NMDS) analysis also revealed that the bacterial communities of the FEA group were separately clustered from the FEB group, and the stress of <0.001 indicated that the NMDS results could accurately reflect the degree of difference from all the samples (Figure S1).

### 3.6 Predicted metabolic pathways and functions of rumen microbiota

**FIGURE 5:**
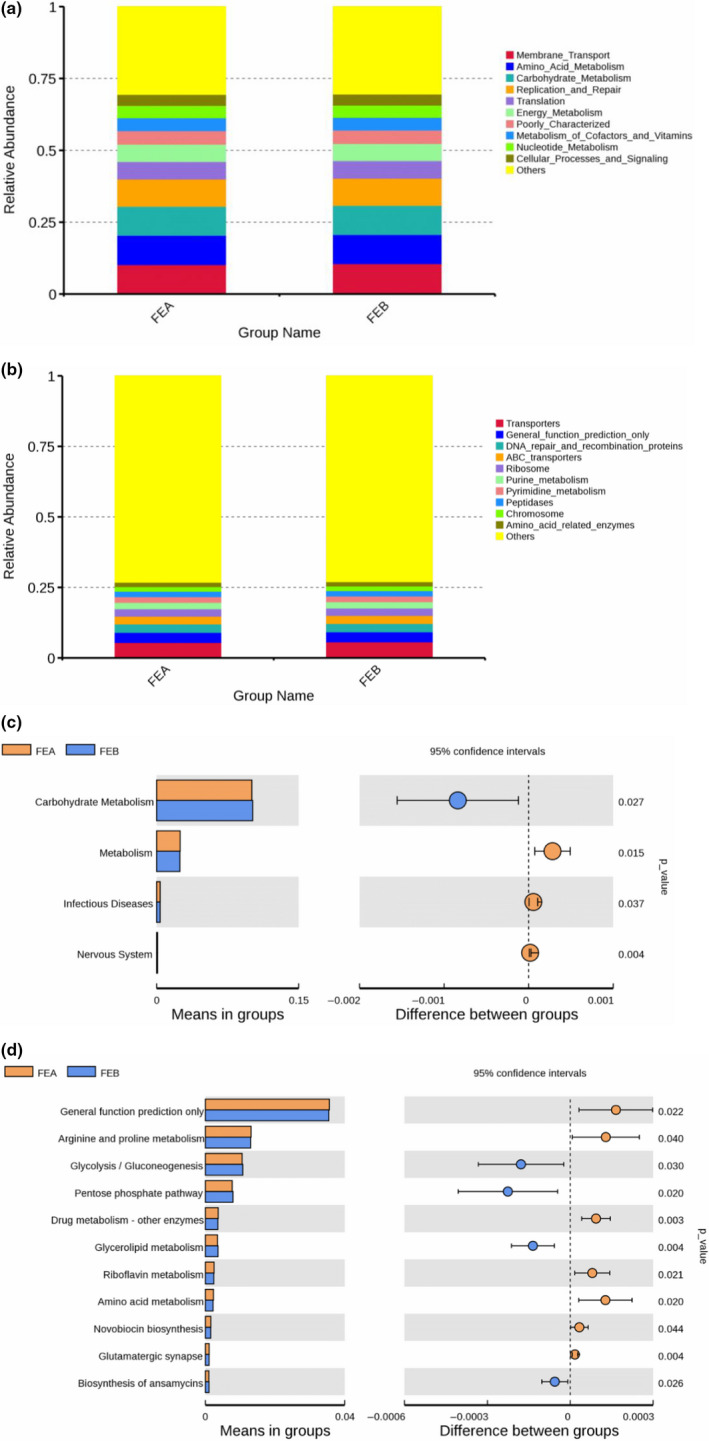
Effects of different dietary energy levels on the predicted functional composition of the rumen bacterial community in female Hu sheep (FEA,9.17 MJ/kg of ME and FEB,10.41 MJ/kg of ME). (a) The majority of the gene sequences annotated to KEGG level 2. (b) The majority of the gene sequences annotated to KEGG level 3. (c) Abundance maps of bacteria with significant differences at KEGG level 2. (d) Abundance maps of bacteria with significant differences at KEGG level 3

The metabolic function of the rumen microbiome was predicted by PICRUSt in the present study. The results of KEGG level 1 showed that “metabolism” was in the highest abundance with more than 47% of the total reads for each group (Figure S2). At KEGG level 2, the 33 gene families of the most abundant (relative abundance > 0.10%) from all rumen samples are presented in Table S3. Genes belonging to membrane transport, amino acid metabolism, carbohydrate metabolism, replication and repair, translation, and energy metabolism had the most relative abundance in the two groups (Figure 5a). Among these gene families, the genes associated with carbohydrate metabolism were dramatically higher (*p* = .027) in the FEB group, and the gene families of metabolism (*p* = .015), infectious diseases (*p* = .037), and nervous system (*p* = .004) were significantly lower in FEB group than those in the FEA group (Figure 5c). At KEGG level 3, the 35 most abundant pathways are shown in Table S3. Among these pathways, transporters, general function prediction only, DNA repair and recombination proteins, ABC transporters, and ribosome were highly represented (Figure 5b).

With an increase in the dietary energy levels, the relative abundance of eleven pathways showed significant variation between the two groups (Figure 5d). The pathways related to glycolysis/gluconeogenesis (*p* = .030), pentose phosphate pathway (*p* = .020), glycerolipid metabolism (*p* = .004), and biosynthesis of ansamycins (*p* = .024) had significant upregulation, whereas the relative abundance of general function prediction only (*p* = .022), arginine and proline metabolism (*p* = .040), drug metabolism‐other enzymes (*p* = .003), riboflavin metabolism (*p* = .021), amino acid metabolism (*p* = .020), novobiocin biosynthesis (*p* = .044), and glutamatergic synapse (*p* = .004) was significantly lower in the FEB group compared with the FEA group.

### 3.7 DISCUSSION

The rumen is the most powerful digestive organ for degrading and converting plant materials to VFAs in ruminants. In this regard, possessing the complex microflora plays a crucial role in feed fermentation and energy metabolism, and more than 70% of the energy is provided by VFAs to ensure host growth and reproduction performance (Flint et al., 2007). Previous studies have demonstrated that the VFA concentration and the proportion of acetate, propionate, and butyrate in the rumen were closely related to the feed type and nutrient level, high energy or concentrate‐based diets tended to increase the VFAs concentration, especially propionate (Agle et al., 2010; Corley & Murphy, 2004; Keady et al., 2001; van Soest., 1994). In the present study, compared with the low energy group, a high energy level significantly increased the molar proportion of butyrate, iso‐valerate, and valerate. These results were similar to that of previous studies (Wang, et al., 2020), in which they found that the proportion of the butyrate, iso‐valerate, and valerate is significantly higher in the high‐concentrate group than those of the high‐forage group.

Previous studies have found that feed conversion efficiency is influenced by dietary components and rumen environment in ruminants, and diet can mainly affect the composition of the rumen microbiome, while the key microbial species may specifically regulate feed efficiency. For instance, *Methanobrevibacter smithii* and *Mitsuokella jalaludinii* may improve ruminal fermentation and further influence feed efficiency (Ellison et al., 2017; Guan et al., 2008; Shabat et al., 2016). Therefore, we speculated that these results may be related to the rumen microbiota and the morphological characteristics of the GIT.

The GIT is the primary site of nutrients for digestion and absorption in ruminants. The GIT tissues are affected by ME intake and dietary energy density. Dietary energy levels can sufficiently promote GIT development and epithelial proliferation, for instance, the high concentrate diet significantly increased the length and width of ruminal papillae, and a decrease in dietary energy decreased the length and width of papillae (Cui et al., 2019; Steele et al., 2016; Wester et al., 1995). The particle size and composition of the diet greatly influenced the morphological structure of rumen papillae (Khan et al., 2011). Previous studies showed that a high energy diet can promote ruminal papillae proliferation in young goats (Shen et al., 2004). In the present study, a high energy diet significantly increased the papillae height compared to the low energy group. These results are consistent with that of previous studies (Kim et al., 2012).

The normal development of small intestinal mucosal structure is the physiological basis of nutrient digestion and absorption. VH, VW, VA, CD, and the ratio of VH/CD are all important histomorphology indicators that reflect intestinal digestion and absorption function (Hedemann et al., 2003; Yang et al., 2016). The change of VH and CD is a positive influence on intestinal epithelial cell proliferation. A higher VH is believed to suggest a greater absorption capacity of nutrients and a deeper CD, a more rapid cell renewal in the villus (Wang et al., 2019). Azim et al. (2011) found that grazing with supplementary concentration can increase the VH of calves. Li et al. (2014) found that nutritional restriction can decrease the CD of jejunum in weaned lambs. Increasing dietary ME can enhance the activity of Na^±^K^+^‐ATPase in small intestinal tissues, which contributes to the hypertrophy and proliferation of normal intestinal epithelial cells, thus promoting the VH in the small intestine of ruminants (Mcleod et al., 2000; Wang et al., 2009). Compared with the low energy group, the VH and CD of duodenal increased significantly in the high energy group, while a tendentious decrease was observed in VH and VH/CD in the jejunum, thereby demonstrating that high energy diet may lead to a negative effect on the jejunum morphology of female *Hu* lambs.

Rumen microbial diversity affects the nutritional health and growth of ruminants and the host provides an appropriate anaerobic environment and fermentation substrates for rumen microorganisms to survive (Guan et al., 2008; Pokharel et al., 2018). In this study, the V4 region of rumen microbial 16S r DNA gene sequencing was sequence to investigate the relationship between rumen microbiota and dietary ME levels in female *Hu* sheep. Previous studies found that a high grain diet can significantly decrease the number of OTUs (Zhang et al., 2017). We observed that the number of OUTs, Chao1 value, and ACE value were indeed significantly decreased in the diet of a high ME level. These results indicated that the relative abundance of rumen microbial communities in female *Hu* lambs is altered by different ME levels in the diets. Moreover, Liu et al. (2013) found that a relatively stable rumen microecological environment and microbial communities may contribute to the absorption and transformation of nutrients in the rumen. Therefore, appropriate dietary energy levels can ensure the normal growth and gastrointestinal health of ruminants.

The composition of rumen microorganisms affects host metabolic function and physiological health. Ye et al. (2016) and Zhang et al. (2017) found that the relative abundance of the dominant microbial phyla is stable in goats. In our research, the dominant three microbial phyla were *Firmicutes*, *Bacteroidetes,* and *Proteobacteria* in the rumen between the low energy and high energy groups, indicating that the rumen microbiota of sheep was also relatively stable at the phyla level. These results are consistent with that of previous studies on cattle (Plaizier et al., 2017; Wetzels et al., 2017). Such studies showed that the most dominant phyla were *Firmicutes* and *Bacteroidetes* in the rumen of ruminants, which were closely related to carbohydrate and protein metabolism (Hook et al., 2011; Yang, et al., 2013). In the present study too, the most dominant phyla in the rumen were *Firmicutes* and *Bacteroidetes* in female *Hu* sheep.

At the genus level, the dominant four genera were *unidentified Ruminococcaceae*, *unidentified Lachnospiraceae*, *unidentified Bacteroidales,* and *Papillibacter*, similar to previous studies (Wang, et al., 2017b; Wang et al., 2016). Notably, it is revealed in our study that the relative abundance of *unidentified Lachnospiraceae* was significantly higher, and *unidentified Ruminococcaceae* too had a higher tendency in the high ME group. Conversely, the relative abundance of *Papillibacter* was very significantly reduced in the high dietary energy level compared with the low dietary energy level. These results indicated that the rumen bacterial dominant genera were dramatically influenced by the energy level of the diet and exhibited great differences among different breeds. The current study also identified the changes of the represented genera in the rumen between the different dietary ME levels. For instance, *unidentified Ruminococcaceae* comprised 5.23%–7.02% of the relative abundance, which was inconsistent with previous studies (Seddik et al., 2018; Wang, et al., 2017). *Ruminococcaceae* strains play a critical role in energy and lipid metabolism, and its relative abundance is negatively associated with vascular sclerosis (Menni et al., 2018). *Unidentified Ruminococcaceae* was the most abundant genus in the two groups in the present study. These results indicated that an appropriate increase in dietary energy levels might contribute to host health in female *Hu* sheep. Additionally, the composition of rumen microbial communities in ruminants may be influenced by breeds, feeding, management, ages, herding way, seasons, and geographic regions (de Menezes et al., 2011). Results of PCoA and NMDS analysis also revealed the distinct bacterial compositions between the low energy group and the high energy group.

The metabolic pathways and functions of rumen microorganisms were predicted by PICRUSt. The relative abundance of predicted functions was detected at KEGG level 2. Among them, membrane transporter was the most abundant pathways associated with environmental information processing, followed by amino acid metabolism, carbohydrate metabolism, replication and repair, translation, and energy metabolism, all of which are essential for the survival, growth, and reproduction of gastrointestinal microbial communities (Lamendella et al., 2011). These results are similar to that of previous studies (Wang, et al., 2017b). As expected, the relative abundance of carbohydrate metabolism in the high energy group was significantly higher than that in the low energy group. At level 3, the relative abundance of transporters was the highest, followed by general function prediction only, DNA repair and recombination proteins, ATP‐binding cassette (ABC) transporter, ribosome, and others. Furthermore, previous studies found that ABC transporters play an important role in the digestion and absorption of nutrients and that ribosomes are closely associated with protein synthesis (Gifford et al., 2013; Yan et al., 2016). The present study showed that the genes responsible for glycolysis/ gluconeogenesis, pentose phosphate pathway, glycerolipid metabolism, and biosynthesis of ansamycins were upregulated in high energy feeding which indicates an enhanced fermentation rate performed by the rumen microbiota, with a decrease in the general function prediction only, arginine and proline metabolism, drug metabolism‐other enzymes, riboflavin metabolism, amino acid metabolism, novobiocin biosynthesis, and glutamatergic synapse‐related genes. These results are inconsistent with that of previous research (Seddik et al., 2018). The current study implied that feeding a high energy diet by using corn as the main energy source and decreasing the roughage percentage alters the ruminal microbial composition and the inferred microbial functions.

## 4 CONCLUSION

The present study mainly studies rumen fermentation, gastrointestinal morphology, and the composition and function of rumen microbiota of growing ewes in *Hu* sheep with different energy feeding. The results suggest that dietary energy levels have major effects on the molar proportion of VFA, morphological structure of GIT, rumen microbial diversity, and inferred metabolic functions. These conclusions provide a significant reference for targeting appropriate dietary energy levels in *Hu* sheep, recommending a high ME level of 10.41MJ/kg in production.

## 5 ANIMAL WELFARE STATEMENT

The authors confirm that the ethical policies of the journal, as noted on the journal's author guidelines page, have been adhered to and the appropriate ethical review committee approval has been received. The authors confirm that they have followed EU standards for the protection of animals used for scientific purposes and feed legislation.

## CONFLICT OF INTEREST

The authors declare that they have no potential conflict of interest.

6

**Table 2:** Effects of different dietary energy levels on rumen fermentation parameters of female Hu lambs

Item	Groups		*SEM*	*p*‐value
	FEA (9.17 MJ/kg)	FEB (10.41 MJ/kg)		
Molar proportion (mol/100mol)				
Acetate	68.85	67.69	0.53	0.301
Propionate	15.72	15.25	0.47	0.642
Butyrate	9.11	9.80	0.16	0.020
Iso‐butyrate	2.23	2.44	0.07	0.169
Iso‐valerate	3.07	3.58	0.12	0.028
Valerate	1.03	1.25	0.04	0.005

**Table 3:** Effects of different dietary energy levels on gastrointestinal histomorphology characteristics of female Hu lambs

Item^a^	Measured index (μm)	Groups		*SEM*	*p*‐value
		FEA (9.17 MJ/kg)	FEB (10.41 MJ/kg)		
Duodenum	Villus height	343.76	445.61	21.42	0.005
	Crypt depth	178.44	267.22	18.69	0.005
	Villus width	131.54	132.42	2.56	0.861
	VH/CD	1.97	1.68	0.09	0.112
Jejunum	Villus height	418.47	325.35	28.21	0.099
	Crypt depth	135.77	154.25	8.47	0.302
	Villus width	128.12	133.32	1.62	0.111
	VH/CD	3.21	2.15	0.30	0.077
Ileum	Villus height	320.17	307.92	17.40	0.747
	Crypt depth	168.22	160.94	10.14	0.742
	Villus width	126.47	132.22	3.20	0.401
	VH/CD	1.96	1.95	0.12	0.973
Rumen	Papillary height	1,486.90	1755.69	63.32	0.020

^a^ VH/CD, Villus height/Crypt depth.

**Table 4:** Diversity indices of ruminal microflora in female Hu lambs fed different dietary energy levels

Item	Groups		*SEM*	*p*‐value
	FEA (9.17 MJ/kg)	FEB (10.41 MJ/kg)		
OTUs^a^	1,105.40	1,050.00	14.04	0.039
Shannon indies^b^	7.82	7.88	0.08	0.712
Simpson indies^b^	0.98	0.98	0.00	0.551
ACE value^c^	1,247.50	1,150.15	24.31	0.035
Chao1 value^c^	1,233.70	1,147.79	17.76	0.005
Goods‐coverage^d^	0.99	0.99	0.00	0.147
PD‐whole‐tree^e^	89.48	86.30	1.09	0.156

^a^ OTU = operational taxonomic unit; Number of operational taxonomic units.

^b^ Shannon and Simpson diversity index.

^c^ ACE = abundance‐based coverage estimator; ACE and Chao species richness estimators.

^d^ Sequencing depth index.

^e^ Phylogenetic diversity index.

## Supporting information

Supplementary Material
