# Supplementary material for: Effects of dietary energy levels on rumen fermentation, microbiota, and gastrointestinal morphology in growing ewes
Source: Food Sci Nutr. 2020 Nov 10;8(12):6621–32. doi: 10.1002/fsn3.1955 (PMC7723210; doi:10.1002/fsn3.1955)
Supplement: Supplementary file 1 — Supplementary Material [file FSN3-8-6621-s001.docx]

**Supplementary Materials**

**Journal: Food Science & Nutrition**

Effects of dietary energy levels on rumen fermentation, microbiota, and gastrointestinal morphology in growing ewes

Qiye Wang^1,3^ | Yancan Wang^1^ | Xin Wang^1^ | Chunpeng Dai^3^ | Jianzhong Li^1^ | Pengfei Huang^1^ | Yali Li^1^ | Xinqin Ding^1^ | Jing Huang^1^ | Tarique Hussain^4^ | Huansheng Yang^1,2,*^ | Mingzhi Zhu^3, *^

**Supplementary Table 1** Overview of species annotation for all samples

| Item | Number or proportion | Dominant species |
| --- | --- | --- |
| OTU catalogue^1^ | 1826 | - |
| Annotated on database | 1826(100.00%) | - |
| Annotated on Unclassified | 0 | - |
| Annotated on Kingdom level | 100.00% | - |
| Annotated on Phylum level | 94.80% | *Firmicutes, Bacteroidete, Euryarchaeota* |
| Annotated on Class level | 92.88% | *Clostridia, Bacteroidia, Negativicutes* |
| Annotated on Order level | 89.43% | *Clostridiales, Bacteroidales, Selenomonadales* |
| Annotated on Family level | 73.17% | *Ruminococcaceae, Christensenellaceae, Rikenellaceae* |
| Annotated on Genus level | 419(22.95%) | *unidentified_Bacteroidales, unidentified_Ruminococcaceae, unidentified_Lachnospiraceae* |
| Annotated on Species level | 8.93% | *Bacteroidales_bacterium*_Bact_22, *rumen_bacterium*_NK4A214, *rumen_bacterium*_YS3 |

OTU = operational taxonomic unit.

^1^Total number of operational taxonomic units for all samples.

**Supplementary Table 2** Variance analysis of taxonomic composition of the 10 most abundance in phylum level and the 30 most abundance in genus level of rumen bacterial community fed different dietary energy levels

| Item | Groups | | SEM | P-value |
| --- | --- | --- | --- | --- |
|  | FEA | FEB |  |  |
| **Phylum level** |  |  |  |  |
| *Firmicutes* | 51.71 | 54.13 | 1.70 | 0.509 |
| *Bacteroidetes* | 43.50 | 41.14 | 1.51 | 0.467 |
| *Tenericutes* | 1.28 | 1.00 | 0.17 | 0.452 |
| *Proteobacteria* | 0.88 | 1.10 | 0.09 | 0.259 |
| *Gracilibacteria* | 0.61 | 0.42 | 0.08 | 0.273 |
| *Spirochaetes* | 0.39 | 0.61 | 0.07 | 0.121 |
| *Euryarchaeota* | 0.19 | 0.31 | 0.06 | 0.344 |
| *Fibrobacteres* | 0.31 | 0.34 | 0.04 | 0.785 |
| *unidentified_Bacteria* | 0.27 | 0.31 | 0.03 | 0.578 |
| *Chloroflexi* | 0.14 | 0.07 | 0.02 | 0.041 |
| Others | 0.70 | 0.57 | 0.04 | 0.143 |
| **Genus level** |  |  |  |  |
| *unidentified_Ruminococcaceae* | 5.23 | 7.02 | 0.54 | 0.096 |
| *unidentified_Bacteroidales* | 4.59 | 3.53 | 0.49 | 0.303 |
| *unidentified_Lachnospiraceae* | 3.41 | 4.70 | 0.29 | 0.012 |
| *Papillibacter* | 2.18 | 1.59 | 0.14 | 0.031 |
| *Saccharofermentans* | 1.58 | 1.57 | 0.16 | 0.970 |
| *Succiniclasticum* | 1.28 | 1.41 | 0.14 | 0.692 |
| *unidentified_Prevotellaceae* | 0.94 | 1.09 | 0.09 | 0.410 |
| *unidentified_Rikenellaceae* | 0.72 | 0.77 | 0.08 | 0.817 |
| *Pseudobutyrivibrio* | 0.40 | 0.52 | 0.09 | 0.581 |
| *unidentified_Clostridiales* | 0.46 | 0.75 | 0.09 | 0.100 |
| *Mycoplasma* | 0.41 | 0.16 | 0.09 | 0.185 |
| *Anaerovorax* | 0.49 | 0.63 | 0.06 | 0.252 |
| *unidentified_Gracilibacteria* | 0.52 | 0.36 | 0.08 | 0.321 |
| *Anaeroplasma* | 0.34 | 0.44 | 0.06 | 0.395 |
| *Methanobrevibacter* | 0.17 | 0.28 | 0.06 | 0.379 |
| *Fibrobacter* | 0.31 | 0.34 | 0.04 | 0.785 |
| *Candidatus_Saccharimonas* | 0.22 | 0.28 | 0.03 | 0.409 |
| *Moryella* | 0.25 | 0.18 | 0.03 | 0.263 |
| *Oribacterium* | 0.20 | 0.29 | 0.03 | 0.089 |
| *unidentified_Spirochaetaceae* | 0.19 | 0.31 | 0.04 | 0.110 |
| *Quinella* | 0.13 | 0.07 | 0.03 | 0.440 |
| *unidentified_Christensenellaceae* | 0.21 | 0.19 | 0.02 | 0.696 |
| *Acetitomaculum* | 0.09 | 0.18 | 0.02 | 0.019 |
| *Desulfovibrio* | 0.16 | 0.19 | 0.02 | 0.486 |
| *Flexilinea* | 0.14 | 0.06 | 0.02 | 0.037 |
| *unidentified_Veillonellaceae* | 0.04 | 0.15 | 0.03 | 0.013 |
| *Desulfobulbus* | 0.02 | 0.09 | 0.02 | 0.105 |
| *Fretibacterium* | 0.09 | 0.05 | 0.02 | 0.222 |
| *Stenotrophomonas* | 0.07 | 0.06 | 0.01 | 0.676 |
| *unidentified_Bacteria* | 0.05 | 0.01 | 0.01 | 0.216 |
| Others | 75.09 | 72.73 | 0.76 | 0.127 |

**Supplementary Table 3** Predicted functions of the rumen bacterial microbiota at level 2 and 3 of female Hu lambs fed different dietary energy levels

| Item | Groups | | SEM | *P-value* |
| --- | --- | --- | --- | --- |
|  | FEA | FEB |  |  |
| **Level 2** |  |  |  |  |
| Environmental Information Processing; Membrane Transport | 10.25 | 10.57 | 0.14 | 0.278 |
| Metabolism; Amino Acid Metabolism | 10.19 | 10.12 | 0.03 | 0.207 |
| Metabolism; Carbohydrate Metabolism | 10.06 | 10.14 | 0.02 | 0.027 |
| Genetic Information Processing; Replication and Repair | 9.50 | 9.47 | 0.03 | 0.530 |
| Genetic Information Processing; Translation | 6.13 | 6.11 | 0.03 | 0.825 |
| Metabolism; Energy Metabolism | 6.02 | 5.96 | 0.03 | 0.396 |
| Unclassified; Poorly Characterized | 4.70 | 4.69 | 0.01 | 0.573 |
| Metabolism; Metabolism of Cofactors and Vitamins | 4.49 | 4.43 | 0.02 | 0.106 |
| Metabolism; Nucleotide Metabolism | 4.27 | 4.24 | 0.02 | 0.394 |
| Unclassified; Cellular Processes and Signaling | 3.85 | 3.85 | 0.00 | 0.551 |
| Cellular Processes; Cell Motility | 2.77 | 2.85 | 0.08 | 0.642 |
| Genetic Information Processing; Transcription | 2.74 | 2.78 | 0.03 | 0.569 |
| Metabolism; Lipid Metabolism | 2.73 | 2.74 | 0.01 | 0.452 |
| Unclassified; Genetic Information Processing | 2.70 | 2.70 | 0.01 | 0.933 |
| Genetic Information Processing; Folding, Sorting and Degradation | 2.54 | 2.51 | 0.02 | 0.393 |
| Unclassified; Metabolism | 2.49 | 2.46 | 0.01 | 0.015 |
| Metabolism; Glycan Biosynthesis and Metabolism | 2.52 | 2.41 | 0.05 | 0.342 |
| Metabolism; Enzyme Families | 2.20 | 2.19 | 0.01 | 0.319 |
| Metabolism; Metabolism of Terpenoids and Polyketides | 1.71 | 1.69 | 0.01 | 0.223 |
| Environmental Information Processing; Signal Transduction | 1.56 | 1.58 | 0.02 | 0.659 |
| Metabolism; Xenobiotics Biodegradation and Metabolism | 1.53 | 1.53 | 0.01 | 0.655 |
| Metabolism; Metabolism of Other Amino Acids | 1.50 | 1.48 | 0.01 | 0.202 |
| Metabolism; Biosynthesis of Other Secondary Metabolites | 1.03 | 1.01 | 0.01 | 0.205 |
| Cellular Processes; Cell Growth and Death | 0.55 | 0.55 | 0.00 | 0.662 |
| Human Diseases; Infectious Diseases | 0.37 | 0.37 | 0.00 | 0.037 |
| Cellular Processes; Transport and Catabolism | 0.36 | 0.33 | 0.01 | 0.310 |
| Organismal Systems; Endocrine System | 0.31 | 0.31 |  | 0.896 |
| Organismal Systems; Environmental Adaptation | 0.16 | 0.16 | 0.00 | 0.239 |
| Environmental Information Processing; Signaling Molecules and Interaction | 0.16 | 0.15 | 0.00 | 0.181 |
| Human Diseases; Metabolic Diseases | 0.11 | 0.11 | 0.00 | 0.671 |
| Human Diseases; Neurodegenerative Diseases | 0.11 | 0.11 | 0.00 | 0.609 |
| Organismal Systems; Nervous System | 0.11 | 0.11 | 0.00 | 0.004 |
| Human Diseases; Cancers | 0.10 | 0.10 | 0.00 | 0.864 |
| **Level 3** |  |  |  |  |
| Environmental Information Processing; Membrane Transport; Transporters | 5.48 | 5.68 | 0.08 | 0.240 |
| Unclassified; Poorly Characterized; General function prediction only | 3.56 | 3.54 | 0.00 | 0.022 |
| Genetic Information Processing; Replication and Repair; DNA repair and recombination proteins | 2.99 | 2.98 | 0.01 | 0.591 |
| Environmental Information Processing; Membrane Transport; ABC transporters | 2.76 | 2.86 | 0.05 | 0.324 |
| Genetic Information Processing; Translation; Ribosome | 2.64 | 2.62 | 0.01 | 0.577 |
| Metabolism; Nucleotide Metabolism; Purine metabolism | 2.25 | 2.24 | 0.01 | 0.543 |
| Metabolism; Nucleotide Metabolism; Pyrimidine metabolism | 2.03 | 2.01 | 0.01 | 0.269 |
| Metabolism; Enzyme Families; Peptidases | 1.95 | 1.93 | 0.01 | 0.225 |
| Genetic Information Processing; Replication and Repair; Chromosome | 1.63 | 1.63 | 0.00 | 0.862 |
| Metabolism; Amino Acid Metabolism; Amino acid related enzymes | 1.56 | 1.55 | 0.00 | 0.266 |
| Genetic Information Processing; Transcription; Transcription factors | 1.46 | 1.51 | 0.03 | 0.367 |
| Genetic Information Processing; Translation; Ribosome Biogenesis | 1.47 | 1.47 | 0.01 | 0.982 |
| Environmental Information Processing; Signal Transduction; Two-component system | 1.42 | 1.45 | 0.02 | 0.636 |
| Metabolism; Carbohydrate Metabolism; Amino sugar and nucleotide sugar metabolism | 1.39 | 1.38 | 0.00 | 0.312 |
| Genetic Information Processing; Replication and Repair; DNA replication proteins | 1.37 | 1.35 | 0.00 | 0.119 |
| Metabolism; Energy Metabolism; Methane metabolism | 1.30 | 1.32 | 0.01 | 0.294 |
| Metabolism; Amino Acid Metabolism; Arginine and proline metabolism | 1.31 | 1.30 | 0.00 | 0.040 |
| Cellular Processes; Cell Motility; Bacterial motility proteins | 1.24 | 1.27 | 0.04 | 0.649 |
| Genetic Information Processing; Translation; Aminoacyl-tRNA biosynthesis | 1.23 | 1.24 | 0.00 | 0.427 |
| Metabolism; Energy Metabolism; Oxidative phosphorylation | 1.24 | 1.20 | 0.01 | 0.202 |
| Environmental Information Processing; Membrane Transport; Secretion system | 1.20 | 1.21 | 0.01 | 0.411 |
| Unclassified; Poorly Characterized; Function unknown | 1.14 | 1.14 | 0.01 | 0.682 |
| Metabolism; Amino Acid Metabolism; Alanine, aspartate and glutamate metabolism | 1.14 | 1.12 | 0.01 | 0.243 |
| Genetic Information Processing; Transcription; Transcription machinery | 1.11 | 1.09 | 0.01 | 0.158 |
| Metabolism; Energy Metabolism; Carbon fixation pathways in prokaryotes | 1.11 | 1.09 | 0.01 | 0.256 |
| Metabolism; Carbohydrate Metabolism; Glycolysis / Gluconeogenesis | 1.05 | 1.07 | 0.00 | 0.030 |
| Unclassified; Cellular Processes and Signaling; Other ion-coupled transporters | 1.06 | 1.05 | 0.00 | 0.373 |
| Metabolism; Carbohydrate Metabolism; Pyruvate metabolism | 1.04 | 1.05 | 0.00 | 0.058 |
| Unclassified; Metabolism; Energy metabolism | 1.05 | 1.02 | 0.01 | 0.133 |
| Genetic Information Processing; Folding, Sorting and Degradation; Chaperones and folding catalysts | 1.04 | 1.03 | 0.01 | 0.381 |
| Genetic Information Processing; Replication and Repair; Homologous recombination | 1.03 | 1.02 | 0.00 | 0.634 |
| Metabolism; Amino Acid Metabolism; Cysteine and methionine metabolism | 1.02 | 1.01 | 0.00 | 0.374 |
| Metabolism; Carbohydrate Metabolism; Starch and sucrose metabolism | 0.99 | 1.00 | 0.00 | 0.306 |
| Unclassified; Genetic Information Processing; Translation proteins | 0.98 | 0.98 | 0.00 | 0.809 |
| Metabolism; Amino Acid Metabolism; Phenylalanine, tyrosine and tryptophan biosynthesis | 0.93 | 0.93 | 0.01 | 0.705 |

**Supplementary Figure 1** NMDS analysis results based on OTU level from different dietary energy levels. Individual samples from MEA1 to MEA5 in group MEA (red); MEB1 to MEB5 in group MEB (blue); MEC1 to MEC5 in group MEC (green).


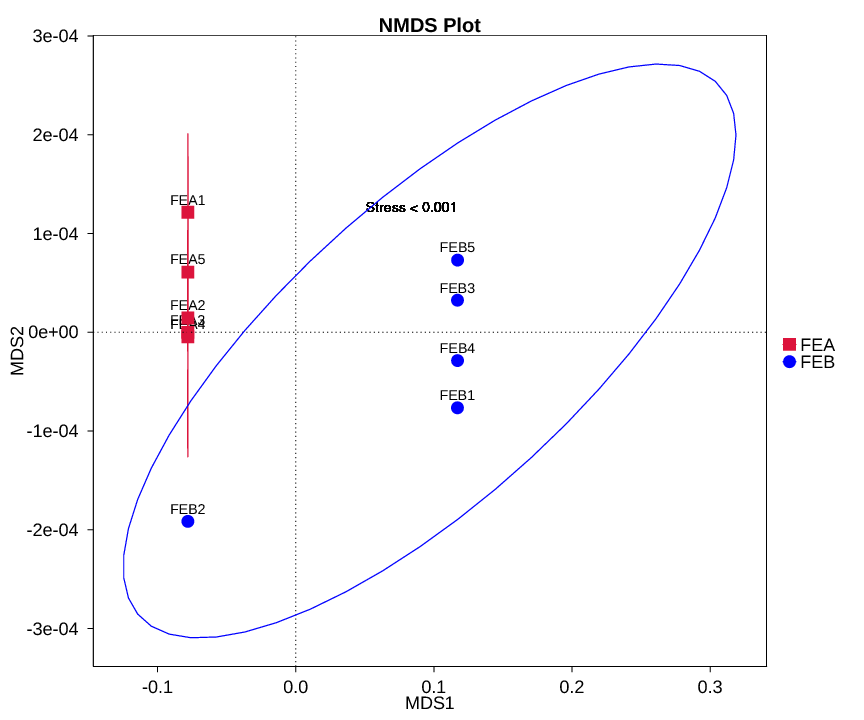


**Supplementary Figure 2** The relative abundance of different functional taxa at level 1, annotated based on the KEGG Orthologs datasets at different dietary nutrient levels
